# Progressive Aortic Regurgitation After Impella Bridge-to-LVAD: A Two-Year Cohort Analysis

**DOI:** 10.3390/biomedicines14030715

**Published:** 2026-03-19

**Authors:** Attila Nemeth, Aron Frederik Popov, Rodrigo Sandoval Boburg, Spiros Lukas Marinos, Helene Häberle, Christoph Salewski, Volker Steger, Christian Schlensak, Medhat Radwan

**Affiliations:** 1 Department of Thoracic and Cardiovascular Surgery, University Hospital Tübingen, 72076 Tübingen, Germany; rodrigo.sandoval-boburg@med.uni-tuebingen.de (R.S.B.); spiros.marinos@med.uni-tuebingen.de (S.L.M.); christoph.salewski@med.uni-tuebingen.de (C.S.); medhat.radwan@med.uni-tuebingen.de (M.R.); 2 Department of Cardiac Surgery, Asklepios Hospital Harburg, 21075 Hamburg, Germany; 3 Department of Anesthesiology and Intensive Care Medicine, University Hospital Tübingen, 72076 Tübingen, Germany; helene.haeberle@med.uni-tuebingen.de

**Keywords:** LVAD, aortic regurgitation, Impella, cardiogenic shock

## Abstract

**Background/Objectives**: Impella support is increasingly utilized as a crucial bridge to durable left ventricular assist device (LVAD) in patients with refractory cardiogenic shock. However, the transvalvular path of the Impella catheter raises concerns regarding mechanical trauma, potentially precipitating or accelerating aortic regurgitation (AR). We aimed to characterize the complete longitudinal trajectory of AR following Impella bridge-to-LVAD and to determine its association with clinical and hemodynamic sequelae. **Methods**: We conducted a single-center retrospective cohort study including all patients bridged from Impella to durable LVAD between 2013 and 2024 (*n* = 19). At Impella initiation, all patients met the retrospective SCAI shock stage D or worse criteria. At LVAD implantation, all patients were classified as INTERMACS 1–2 (INTERMACS 2, *n* = 13). The Impella models were 5.0 in 11 (axillary access), 2.5 in 5 (femoral access), and CP in 3 (femoral access); no periprocedural Impella complications were recorded. The implanted LVAD systems were HeartMate II (*n* = 7), HVAD (*n* = 3), and HeartMate III (*n* = 9). Patients undergoing concomitant aortic valve intervention were excluded. Transthoracic/TEE echocardiography was performed at prespecified time points (pre-Impella, pre-LVAD, post-LVAD discharge, 12 months, and 24 months) with standardized aortic regurgitation (AR) grading. Right ventricular (RV) function was assessed qualitatively when quantitative indices (TAPSE) were unavailable. Primary endpoints were new or progressive AR and AR severity at LVAD implantation. Secondary endpoints included survival, renal dysfunction, biomarkers, and rehospitalization. Univariate analyses were used to compare outcomes according to AR severity. **Results**: Nineteen patients (68% male, median age 57 years, IQR 47–60) underwent Impella support for 13.3 ± 9.9 days before HeartMate 3 (84%) or HVAD (16%) implantation. All patients had competent aortic valves (grade 0 AR) at the time of LVAD implantation. AR ≥ mild developed in 9/18 (50%) at discharge, 12/15 (80%) at 12 months, and 13/15 (87%) at 24 months, and 8/15 (53%) progressed to ≥ moderate AR by 24 months. Patients with moderate-to-severe AR had higher NT-proBNP levels at 12 months (median 6318 vs. 2336 pg/mL, *p* = 0.137). Thirty-day and 24-month survival rates were 95% and 79%, respectively. **Conclusions**: Aortic regurgitation frequently develops or progresses from the pre-LVAD period to follow-up in patients bridged from Impella to durable LVAD. Although limited by a small sample size and incomplete quantitative RV metrics, these observations support structured echocardiographic surveillance after Impella use and management strategies—routine valve inspection at LVAD implantation and post-LVAD speed/blood pressure targets that encourage aortic valve opening—to mitigate the risk and clinical impact of aortic regurgitation.

## 1. Introduction

Temporary mechanical circulatory support is essential for managing cardiogenic shock, with the Impella pump serving as an established bridge to durable left ventricular assist device (LVAD) implantation [1,2]. While Impella-mediated left ventricular unloading improves systemic perfusion, its transvalvular positioning inevitably causes mechanical trauma to the native aortic valve [3,4]. Contemporary data demonstrate that up to 33% of Impella-supported patients develop new or worsening aortic regurgitation (AR) within days to weeks, including those undergoing only high-risk percutaneous coronary intervention or brief hemodynamic support [5,6].

In LVAD recipients, AR is both common and clinically significant, with registry data showing 30–50% develop at least moderate regurgitation within 12–18 months of support. Pathological mechanisms include sustained leaflet tethering, commissural fusion, and aortic sinus dilatation, which are exacerbated by persistent transvalvular gradients from either Impella or continuous-flow LVAD support that keeps the valve closed [7,8,9]. Established AR creates a regurgitant circuit that recycles LVAD output into the left ventricle, increasing left-sided filling pressures and reducing systemic flow, thereby precipitating right ventricular failure and late hemodynamic complications [7,10]. Large LVAD registries have confirmed that moderate-to-severe AR correlates with increased rehospitalizations and mortality, although prior exposure to temporary axial-flow support is rarely documented [9,10]. Evidence linking pre-LVAD Impella support to AR evolution and clinical outcomes remains limited to small, single-center studies with few patients. In a recent study, Lewin et al. reported that preoperative microaxial flow pump support did not significantly affect late AR development after LVAD implantation. However, their analysis was limited by shorter follow-up and different patient selection criteria. Our study builds on these observations by offering comprehensive longitudinal echocardiographic data over a 24-month period in a cohort of severely ill patients (SCAI shock stage D or worse) [11].

We conducted a single-center analysis of all patients who received Impella support before LVAD implantation over the past decade. By tracking the complete valve trajectory from the pre-Impella baseline through peri-implantation and into mid-term LVAD support, we characterized the incidence and severity of de novo or progressive AR that emerged after temporary Impella support.

Our primary aims were to (1) characterize the temporal trajectory of AR severity in this high-risk population and (2) identify early clinical or hemodynamic factors associated with AR progression to ≥moderate severity during two years of LVAD support. We hypothesized that Impella-induced valvular trauma, amplified by LVAD support, would result in a high incidence of clinically significant AR.

## 2. Materials and Methods

### 2.1. Study Design and Setting

We conducted a single-center, retrospective cohort study at the University Hospital of Tübingen, Germany, a tertiary referral center for mechanical circulatory support (MCS). All consecutive adults (≥18 years) who received percutaneous Impella support for cardiogenic shock followed by LVAD implantation between 1 January 2013 and 31 December 2024, were included. The sample size was limited by the available cohort meeting the inclusion criteria during the study period. No formal power calculation was performed for this study. The local Ethics Committee approved the study protocol (approval no. 440/2020B) in accordance with the Declaration of Helsinki. Individual informed consent was waived due to the exclusive use of de-identified data.

### 2.2. Patient Population

We included patients ≥18 years of age who presented with cardiogenic shock and met the criteria for retrospective SCAI Shock Stage D or worse at the time of Impella insertion. At LVAD implantation, all patients were categorized as INTERMACS profiles 1 or 2 (requiring continuous urgent support). Impella support was used as a bridge to single left ventricular continuous-flow LVAD implantation. Complete transthoracic echocardiography (TTEs) was mandatory for inclusion. We excluded patients with biventricular Impella configurations, postcardiotomy or post-transplant shock, prior heart transplantation, or missing or nondiagnostic echocardiograms. Pre-existing AR of any grade was not exclusionary, as we aimed to track the complete AR trajectory. The results were compared with those of contemporary LVAD cohorts without prior Impella exposure.

All patients received percutaneous Impella support (Abiomed, Danvers, MA, USA) via femoral or axillary access according to clinical indications. Standard institutional anticoagulation protocols were followed. Pump speed adjustments were made based on clinical assessment without standardized protocols for aortic valve opening. The Impella duration was minimized when feasible. LVAD implantation (HeartMate 3 [Abbott, Abbott Park, IL, USA] or HVAD [Medtronic, Minneapolis, MN, USA]) was performed via a median sternotomy without concomitant valve intervention.

### 2.3. Data Collection

Comprehensive clinical data, including baseline hemodynamics (mean arterial pressure), metabolic markers (pH), key echocardiographic parameters (LVEF), and renal function markers (creatinine), were collected at prespecified time points. While extraction of highly granular markers (such as lactate, central venous pressure, and mixed venous saturation) was attempted, these were ultimately excluded from the final comparative analysis due to a high degree of missingness inherent to the retrospective chart review. Right ventricular function was primarily assessed using Tricuspid Annular Plane Systolic Excursion (TAPSE) where available, supplemented by qualitative assessment per institutional protocol.

Transthoracic echocardiograms (TTEs) were obtained at five time points: (1) baseline, within 24 h before Impella insertion; (2) pre-LVAD, within 72 h before LVAD implantation; (3) early post-LVAD, within 72 h before discharge; (4) 12-month follow-up; and (5) 24-month follow-up. All TTEs were performed using a Philips CX50 ultrasound system (Hamburg Germany), archived in the DICOM format, and interpreted on-site by attending physicians.

Aortic regurgitation was graded according to the German Society for Ultrasound in Medicine (DEGUM) guidelines using the color Doppler jet width-to-left ventricular outflow tract diameter ratio and pressure half-time measurements. The severity was classified as none, mild, moderate, or severe. Observer variability was not assessed.

### 2.4. Outcomes

The primary endpoints were (1) incidence of new or progressive AR after Impella support, defined as the first TTE showing at least mild regurgitation, and (2) AR severity at LVAD implantation. The secondary endpoints included overall survival at 30 days, 6 months, and 12 months; postoperative renal insufficiency (serum creatinine > 2.0 mg/dL or ≥2-fold baseline increase); hemolysis and ventricular unloading markers (peak LDH and NT-proBNP at discharge, 6 months, and 12 months); and unplanned rehospitalizations within 24 months. LVAD performance metrics were excluded because of confounding factors such as speed modulation, right ventricular function, and volume status.

### 2.5. Statistical Analysis

Statistical analyses were performed using IBM SPSS Statistics (version 29). Normality was assessed using the Shapiro–Wilk test. Continuous variables are presented as mean ± SD for normally distributed data or median [IQR] for non-normally distributed data. Comparisons between groups were performed using unpaired *t*-tests (normal) or Mann–Whitney *U* tests (non-normal). Categorical variables were compared using Fisher’s exact test, given the small sample size. Given the limited sample size (*n* = 19), analyses were restricted to univariate comparisons only, without multivariable modeling, to prevent overfitting and spurious conclusions. No adjustments for multiple comparisons were performed in this exploratory analysis. Statistical significance was set at *p* < 0.05 (two-sided). Missing data were handled using complete case analysis, with missingness ≤5% per variable.

## 3. Results

### 3.1. Patient Cohort

From January 2013 to December 2024, 19 consecutive patients underwent Impella support bridging to LVAD implantation (Table 1 and Table S1). The median age was 57 years (IQR 47–60), 68% were male, and the median BMI was 27.7 kg/m^2^ (IQR 23.3–30.6). The etiology of cardiogenic shock was ischemic cardiomyopathy in 11 patients (58%) and dilated cardiomyopathy in eight (42%).

Impella CP, 2.5, and 5.0 devices were used in three (16%), five (26%), and 11 (58%) patients, respectively. The mean Impella support duration was 13.3 ± 9.9 days. LVAD implantation included HeartMate 3 in 16 patients (84%) and HVAD in 3 (16%).

No operative deaths were observed. Survival was 95% (*n* = 18) at 30 days, 89% at 6 months, and 79% at 1 and 2 years. No patient required Impella reimplantation or LVAD exchange.

### 3.2. Incidence and Progression of Aortic Regurgitation

**Figure 1:**
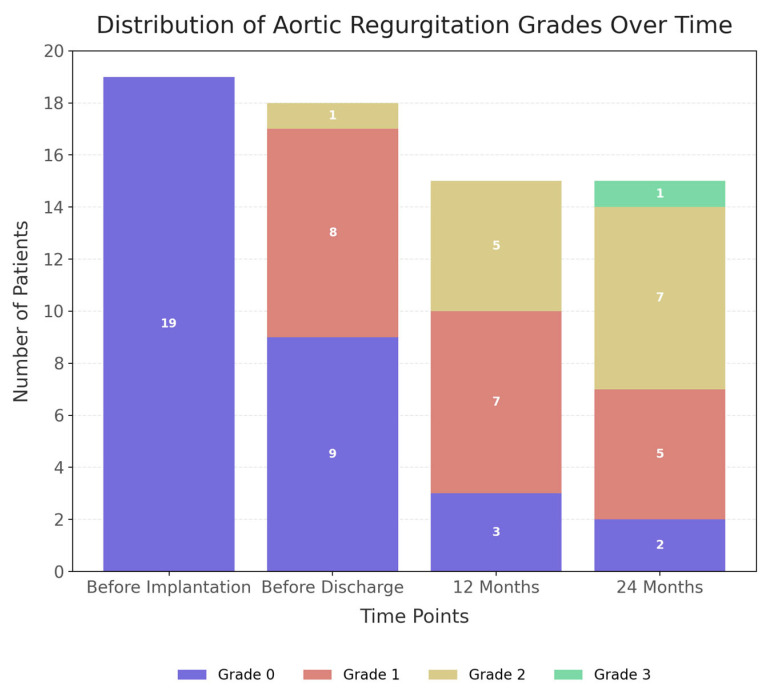
Temporal Progression of Aortic Regurgitation Severity in Patients Undergoing Impella Support and Subsequent LVAD Implantation.

The primary finding of this study was the rapid and persistent progression of AR. Figure 1 shows that all the aortic valves were competent (grade 0) at the time of LVAD implantation. By the time of hospital discharge, AR (≥grade 1) had developed in half of the cohort (nine patients, 50%). This progression accelerated during follow-up: at 12 months, 80% of patients had ≥mild AR, and by 24 months (15 patients at risk), 13 patients (87%) had at least mild AR, with over half (8 patients, 53%) progressing to ≥moderate AR (grade 2 or 3). Only two patients (13%) remained regurgitation-free at this time point. This highlights the delayed onset of clinically relevant valvular dysfunction. Freedom from at least mild AR decreased from 100% preoperatively to 50% at discharge, 20% at 12 months, and 13% at 24 months.

### 3.3. Laboratory Parameters and Biomarkers

NT-proBNP showed wide dispersion with median values of 1547 pg/mL (IQR 4462) and 1774.5 pg/mL (IQR 2634.5) at 12 and 24 months, respectively. Serum creatinine remained stable at a median of 1.05 mg/dL (IQR 0.43) at 6 months, 1.20 mg/dL (IQR 0.70) at 12 months, and 1.20 mg/dL (IQR 0.39) at 24 months. LDH decreased from 255 U/L (IQR 113) at discharge to 198 U/L (IQR 70) at 6 months and 209 U/L (IQR 67.5) at 12. The median LDH level at 24 months was 200 U/L (IQR 91).

### 3.4. Prognostic Factors for the Development of AR

For any AR (≥grade 1) at 24 months, significant associations were observed with elevated NT-proBNP levels at 12 (*p* = 0.019) and 24 months (*p* = 0.044) and higher creatinine levels at 6 months (*p* = 0.032). For moderate or greater AR (≥grade 2), NT-proBNP remained the strongest, albeit non-significant, association at 12 months (median 4057 vs. 878 pg/mL, *p* = 0.072). Furthermore, a trend suggested that reduced Tricuspid Annular Plane Systolic Excursion (TAPSE) at 24 months (median 13.0 vs. 14.0 mm, *p* = 0.167) and lower BMI (*p* = 0.094) were associated with ≥grade 2 AR. Importantly, univariate analysis (Table 2) found no significant association between Impella duration, Impella type, or ECLS co-support and AR development at any grade.

## 4. Discussion

In this single-center retrospective cohort study, we examined the incidence and evolution of AR in patients who underwent Impella support before LVAD implantation. Our data demonstrate that despite the absence of AR at the time of LVAD implantation, 50% of patients developed at least mild AR by the time of hospital discharge, and over half (53%) progressed to moderate or greater AR within 24 months of follow-up period. These findings underscore the clinical relevance of Impella-induced valvular trauma, even when the initial echocardiographic assessment appears benign. This trajectory of progressive deterioration, identified through longitudinal tracking, was the central observation of our study.

The pathophysiology underlying the development of AR in this context likely reflects mechanical trauma to the aortic valve caused by the transvalvular placement of the Impella catheter. Studies such as those by Hironaka et al. and Butala et al. have reported structural changes, including leaflet prolapse, cusp indentation, and fibrosis, following Impella support, particularly in longer durations exceeding 7–10 days [5,12]. Our mean support duration of 13.3 days significantly exceeded the 7-day threshold previously identified as a risk factor for structural leaflet changes. This suggests that for the ‘Bridge-to-LVAD’ population, valvular trauma may be an almost universal sequela of the support duration required for clinical stabilization.

Our observations are in line with those of Rao et al., who found a disproportionately high rate of de novo AR in patients bridged with Impella before LVAD compared to those receiving other temporary support modalities [4]. However, our study extends these findings by demonstrating the progressive deterioration of valve function over a 2-year follow-up period, suggesting that subclinical valvular injury from Impella use may evolve into clinically significant regurgitation over time. On the other hand, Lewin et al. recently observed that preoperative microaxial flow pump support did not have a significant impact on the progression of late AR. Nonetheless, the variation in patient acuity—our study focused exclusively on individuals at SCAI stage D or worse—combined with the extended follow-up period in our research and the possibility of survivor bias, might account for these differing findings. Similar trajectories were noted in the INTERMACS registry, where Truby et al. reported a progressive rise in moderate-to-severe AR in LVAD patients, particularly in those with closed aortic valves and prior device support [9].

From a clinical standpoint, the presence of moderate or greater AR after LVAD implantation is associated with impaired systemic flow, increased left ventricular filling pressure, and a heightened risk of right ventricular failure [13,14]. In our cohort, AR progression was associated with increased rehospitalization and showed non-significant trends with adverse biomarker profiles (NT-proBNP) and reduced RV function (TAPSE). Given the small sample size, these univariate associations should be cautiously interpreted as exploratory findings. It is important to note that statistical significance does not necessarily equate to clinical significance in this small cohort. However, the marked elevation in NT-proBNP (median 4057 pg/mL for the high AR group) is consistent with volume overload and adverse remodeling that characterizes the hemodynamic burden of significant AR in the setting of continuous-flow LVAD support. Notably, no patients in our cohort required surgical aortic valve intervention during the 24-month follow-up period. Notably, Rubinstein et al. showed that patients with moderate AR exhibit increased rates of late right heart failure and require more frequent pump speed adjustments to maintain hemodynamic targets [13].

Given the implications for patient outcomes, our findings support a more aggressive approach to aortic valve surveillance and management in patients bridged with Impella. Intraoperative aortic valve inspection at the time of LVAD implantation should be considered standard practice. In selected cases with even mild pre-existing AR or prolonged Impella exposure, concomitant aortic valve closure (e.g., Park stitch) or bioprosthetic replacement may be warranted, as previously advocated by Oishi et al. and Ueda et al. [15,16]. While surgical risk must be balanced against the hemodynamic burden of AR, our results suggest that early intervention may mitigate progressive deterioration and downstream right-heart dysfunction.

## 5. Conclusions

Among patients with profound cardiogenic shock successfully bridged from Impella support to durable LVAD implantation, the aortic valve is highly susceptible to mechanical injuries. Our findings indicate that the development of new or worsening aortic regurgitation is a common late-onset issue, occurring in almost 90% of patients and progressing to clinically significant (≥moderate) AR in over half (53%) within two years of follow-up. Although our small, exploratory cohort precludes definitive multivariable risk stratification, the observed trends linking AR progression with elevated NT-proBNP and reduced RV function suggest a detrimental hemodynamic impact. Although recent data from Lewin et al. indicate that microaxial flow pump support may not independently influence the development of late AR, the high incidence observed in our severely ill cohort necessitates ongoing vigilance. These findings necessitate structured, long-term echocardiographic monitoring and advocate for a low threshold in adopting pragmatic management strategies. This includes routine intraoperative aortic valve inspections and post-LVAD speed adjustments to promote valve opening and reduce the risk of AR progression in this high-risk patient cohort.

## 6. Limitations

Our study had several limitations. As a retrospective, single-center analysis, it is inherently subject to selection bias and has limited generalizability. Although echocardiographic assessments were standardized using the DEGUM guidelines, the absence of centralized core lab adjudication introduces the potential for observer variability. The retrospective design resulted in incomplete granular hemodynamic and metabolic data during the Impella support phase, limiting our ability to correlate specific continuous pump metrics or varying shock severities directly with AR development. Additionally, our sample size (*n* = 19) precludes robust multivariable modeling and may limit the detection of subtle associations with the clinical outcomes. AR jet morphology (central vs. eccentric) was not systematically recorded, which limits our ability to elucidate the precise mechanism of valvular injury. Furthermore, the absence of a matched control group of LVAD recipients without prior Impella support precludes definitive attribution of AR progression to Impella-specific injury versus the natural history of AR under continuous-flow LVAD support. The analysis of factors associated with AR ≥ grade 1 at 24 months is limited by the small number of patients without AR (*n* = 2), which reduces statistical power and should be interpreted with caution.

Future prospective studies with larger cohorts are needed to validate these findings. The integration of serial advanced imaging techniques and standardized AR quantification protocols, including systematic characterization of AR jet morphology, may also improve risk stratification and therapeutic decision-making in this high-risk population.

## Figures and Tables

**Table 1:** Baseline Demographics, Etiology, Device Characteristics, and Survival Outcomes of Patients Receiving Impella Support Prior to LVAD Implantation (*n* = 19).

Variable	Overall (*n* = 19)
**Demographics**	
Age, years (median, IQR)	57 (47–60)
Male (%)	13 (68)
BMI (median, IQR)	27.7 (23.3–30.6)
**Etiology of Cardiogenic Shock**	
Ischemic Cardiomyopathy (%)	11 (58)
Dilatative Cardiomyopathy (%)	8 (42)
**Impella Type**	
CP (%)	3 (16)
2.5 (%)	5 (26)
5.0 (%)	11 (58)
**Impella Support Duration (days)**	13.3 (±9.9)
**Length of Stay (days)**	68.7 ± 25.7 days
**CPR before Implantation (%)**	2 (11)
LVAD Type	
HVAD (%)	3 (16)
HeartMate III (%)	16 (84)
**MAP before Implantation (mmHg)**	78 ± 34
**pH before Implantation**	7.45 ± 0.06
**Creatinine before Implantation (mg/dL)**	1.20 ± 0.58
**Survival**	
30-Day Survival (%)	18 (95)
6-Month Survival (%)	17 (89)
1-Year Survival (%)	15 (79)
2-Year Survival (%)	15 (79)

**Table 2:** Univariate comparison of baseline and follow-up variables between patients who did or did not develop high-grade aortic regurgitation (AR ≥ grade 2).

Predictor	*p*-Value	Effect Size ^†^	Median (No High AR)	Median (High AR)
Body-mass index (kg m^−2^)	0.442	0.252	27.778	25.438
Impella support duration (days)	0.679	0.301	12	15
LDH at discharge (U L^−1^)	0.203	0.858	236.5	314
BNP at 12 months (pg mL^−1^)	0.072	0.658	878	4057
Creatinine at 6-month follow-up (mg dL^−1^)	0.267	0.220	0.95	1.10

^†^ Effect size calculated as absolute standardized mean difference.

## Data Availability

The data supporting the findings of this study are available from the corresponding author upon reasonable request. Access is restricted because the underlying database contains additional, currently unpublished observations earmarked for ongoing and future research projects.

## Supplementary Materials

The following supporting information can be downloaded at: https://www.mdpi.com/article/10.3390/biomedicines14030715/s1, Table S1: Individual Patient Data.

## Abbreviations

The following abbreviations are used in this manuscript:

AR	Aortic Regurgitation
BMI	Body Mass Index
DEGUM	German Society for Ultrasound in Medicine
DICOM	Digital Imaging and Communications in Medicine
ECLS	Extracorporeal Life Support
HVAD	HeartWare Ventricular Assist Device
INTERMACS	Interagency Registry for Mechanically Assisted Circulatory Support
IQR	Interquartile Range
LVAD	Left Ventricular Assist Device
MCS	Mechanical Circulatory Support
NT-proBNP	N-terminal pro-brain natriuretic peptide
LDH	Lactate Dehydrogenase
TAPSE	Tricuspid Annular Plane Systolic Excursion
TTE	Transthoracic Echocardiography
SD	Standard Deviation
