# Supplementary material for: Progressive Aortic Regurgitation After Impella Bridge-to-LVAD: A Two-Year Cohort Analysis
Source: Biomedicines. 2026 Mar 19;14(3):715. doi: 10.3390/biomedicines14030715 (PMC13024325; doi:10.3390/biomedicines14030715)
Supplement: Supplementary file 1 [file biomedicines-14-00715-s001.zip › biomedicines-4153948-supplementary.pdf]

biomedicines

MDPI

**Table S1.** Individual Patient Data.

| Patient | Age (years) | Sex | Impella Type | Impella Duration (days) | LVAD Type | ECLS Therapy | 24 Months Follow-Up Status |
|---|---|---|---|---|---|---|---|
| 1 | 51 | Male | 5 | 33 | Heartmate III | Yes | Alive |
| 2 | 62 | Male | 5 | 19 | Heartmate III | No | Alive |
| 3 | 48 | Female | CP | 24 | Heartmate III | Yes | Alive |
| 4 | 50 | Male | 5 | 18 | Heartmate III | No | Alive |
| 5 | 62 | Male | 2,5 | 4 | Heartmate III | Yes | Alive |
| 6 | 20 | Female | 2,5 | 5 | Heartmate III | Yes | Lost to follow-up |
| 7 | 59 | Male | 5 | 29 | Heartmate III | Yes | Alive |
| 8 | 11 | Female | 2,5 | 2 | Heartware | Yes | Lost to follow-up |
| 9 | 35 | Female | 5 | 8 | Heartware | Yes | Alive |
| 10 | 61 | Male | 5 | 20 | Heartware | Yes | Alive |
| 11 | 67 | Male | CP | 4 | Heartmate III | Yes | Deceased |
| 12 | 46 | Male | 5 | 0 | Heartmate III | Yes | Alive |
| 13 | 57 | Male | 5 | 12 | Heartmate III | No | Alive |
| 14 | 73 | Male | 5 | 0 | Heartmate III | No | Alive |
| 15 | 59 | Female | 5 | 14 | Heartmate III | Yes | Lost to follow-up |
| 16 | 57 | Male | CP | 18 | Heartmate III | No | Alive |
| 17 | 47 | Female | 2,5 | 6 | Heartmate III | Yes | Alive |
| 18 | 57 | Male | 2,5 | 12 | Heartmate III | Yes | Alive |
| 19 | 47 | Male | 5 | 24 | Heartmate III | Yes | Alive |
